# Building Influenza Surveillance Pyramids in Near Real Time, Australia

**DOI:** 10.3201/eid1911.121878

**Published:** 2013-11

**Authors:** Craig B. Dalton, Sandra J. Carlson, Michelle T. Butler, Elissa Elvidge, David N. Durrheim

**Affiliations:** Hunter New England Population Health, Newcastle, New South Wales, Australia (C.B. Dalton, S.J. Carlson, M.T. Butler, E. Elvidge, D.N. Durrheim);; Newcastle University, Newcastle (C.B. Dalton, D.N. Durrheim)

**Keywords:** influenza, epidemiology, public health, population surveillance, data collection, viruses

## Abstract

A timely measure of circulating influenza virus severity has been elusive. Flutracking, the Australian online influenza-like illness surveillance system, was used to construct a surveillance pyramid in near real time for 2011/2012 participants and demonstrated a striking difference between years. Such pyramids will facilitate rapid estimation of attack rates and disease severity.

Data from several influenza surveillance systems are integrated in Australia each year (*1*–*3*) to create a timely and accurate picture of influenza activity. Each surveillance method has its strengths and limitations. The online national Flutracking surveillance system contributes to Australian influenza surveillance by providing weekly community-level influenza-like illness (ILI) attack rates not biased by health-seeking behavior and clinician-testing practices (*4*–*7*). The Flutracking surveillance system has been incorporated into the weekly national Australian influenza report since 2009 (*3*) to 1) compare ILI syndrome rates of vaccinated and unvaccinated participants to detect interpandemic and pandemic influenza and provide early confirmation of vaccine effectiveness or failure; 2) provide consistent surveillance of influenza activity across all jurisdictions and over time; and 3) enable year-to-year comparison of the timing, incidence, and severity of influenza.

In 2011, new questions were added to the Flutracking surveillance system to document health-seeking behavior and laboratory confirmation of influenza infection among participants. This enabled regular timely calculation of influenza surveillance pyramids to examine the proportion of participants with ILI that sought medical care, the type of medical care sought, and the proportion tested for, and confirmed to have, influenza infection. Surveillance pyramids provide a model for estimating the relative attrition as patients transition the multiple steps for an episode of illness to be registered in surveillance data (*8*). Flutracking data for 2011 and 2012 were used to investigate whether a near real time severity measure for circulating influenza strains could be determined.

## The Study

The Flutracking surveillance system was in operation for 24 weeks in 2011 from the week ending May 8 to the week ending October 16, and 24 weeks in 2012 from the week ending May 6 to the week ending October 14. Recruitment methods in 2011 and 2012 were similar to those used in 2007–2010 (*4*).

The weekly survey questions in 2011 and 2012 were similar to those used in 2007–2010 (*4*). However, in 2011, the following questions were added to the weekly questionnaire:

Did participants reporting cough and fever seek health advice because of their illness? Response options for type of advice sought included an emergency department/after-hours service, general practitioner, 24-hour health advice telephone hotline, advice from other medical professional, or admitted as a hospital inpatient. Did a doctor or nurse tell the participant, who sought health advice, that they had influenza or another illness? Did you have an influenza test (for those who sought health advice)? If so, was it positive for influenza?

We compared participation numbers from 2006 through 2012 at national level. Surveillance pyramids were then produced for 6-week blocks for the weeks ending as follows: in 2011, May 8–June 12, June 19–July 24, July 31–September 4, and September 11–October 16; and in 2012, May 6–June 10, June 17–July 22, July 29–September 2, and September 9–October 14. The pyramid base comprised the number of participants reporting fever and cough over the 6-week period; the next layer was the subset of participants who sought medical advice (from a general practitioner, emergency department/after-hours service, as a hospital inpatient,). The next layer was the number of participants who reported having a laboratory test for influenza or a positive influenza laboratory test result over the 6-week period. We used these pyramids to estimate the relationship between ILI at the community level and national influenza laboratory reports. In addition, we calculated the weekly percentage of participants in 2011 and 2012 who had fever and cough and >2 days off from work or normal duties, as well as the weekly percentage of participants in 2011 and 2012 who visited a general practitioner or emergency department or stayed in a hospital because of fever and cough.

The number of participants who had completed at least 1 survey increased from 394 in 2006, to 982 in 2007, 4,827 in 2008, 8,546 in 2009, 12,581 in 2010, 13,101 in 2011, and 16,046 in 2012. Among the 12,109 participants in 2011 and 14,467 participants in 2012 who completed at least 1 survey in the first 4 weeks of the survey each year, the median weekly participation rate for the remainder of each year was 95.8%. Of the 318,302 surveys completed in 2012, participants reported 10,379 (3.3%) episodes of fever and cough, and among 263,778 surveys completed in 2011, there were 8,009 (3.0%) reported episodes of fever and cough. Those who experienced the 8,009 episodes also reported 2,409 (30.1%) visits to general practitioners along with 184 (2.3%) visits to other health professionals, 142 (1.8%) visits to emergency departments, 45 calls (0.6%) to 24-hour advice lines, and 39 (0.5%) stays in the hospital.

In 2012, among 10,379 episodes of fever and cough reported by Flutracking participants, participants reported 3,170 (30.5%) visits to general practitioners, 202 (1.9%) visits to other health professionals, 189 (1.8%) visits to emergency departments, 69 (0.7%) calls to 24-hour advice lines, and 37 (0.4%) stays in the hospital. In 2011, the proportion of participants with fever and cough, who also sought medical advice and had a positive laboratory test, was highest during September 11–October 16. During this period, 34.4% (573/1,665) of participants sought medical advice for their symptoms, and 4.5% (26/573) of participants who sought medical advice had a laboratory test for influenza, of whom 50.0% (13/26) reported having a positive influenza test result. 

In 2012, the proportion of participants with fever and cough, who sought medical advice and had a positive laboratory test result, was highest during July 29–September 2. During this period, 34.5% (1,054/3,059) of participants sought medical advice for their symptoms, and 8.6% (91/1,054) of participants who sought medical advice had a laboratory test for influenza, of whom 35.2% (32/91) reported having a positive influenza test result (Table). Compared with 2011 participants, a higher weekly percentage of participants in 2012 took >2 or days off from work, visited general practitioners or emergency departments, and stayed in the hospital because of fever and cough (Figure).

**Table:** Comparison of 6 weekly Flutracking surveillance pyramid results, Australia, 2011 and 2012*

Participant characteristic	Weeks ending, no. (%)							
	May 8–Jun 12, 2011	May 6–Jun 10, 2012	Jun 19–Jul 24, 2011	Jun 17–Jul 22, 2012	Jul 31–Sep 4, 2011	Jul 29–Sep 2, 2012	Sep 11–Oct 16, 2011	Sep 9–Oct 14, 2012
Positive laboratory test result	9 (0.5)	4 (0.2)	8 (0.4)	21 (0.6)	15 (0.7)	32 (1.1)	13 (0.8)	10 (0.6)
Laboratory test for influenza	24 (1.2)	26 (1.1)	38 (1.8)	35 (1.1)	28 (1.2)	91 (3.0)	26 (1.6)	21 (1.3)
Sought medical advice (GP/ED/inpatient)	569 (28.9)	679 (28.5)	690 (32.4)	1,052 (32.0)	698 (31.1)	1,054 (34.5)	573 (34.4)	521 (31.6)
Reported fever and cough	1,967 (100.0)	2,380 (100.0)	2,131 (100.0)	3,289 (100.0)	2,246 (100.0)	3,059 (100.0)	1,665 (100.0)	1,651 (100.0)
No. surveys completed	64,869	77,235	67,612	81,365	67,006	81,385	64,290	78,317
Ratio of positive laboratory test results to cough and fever	1:218	1:595	1:266	1:157	1:150	1:96	1:128	1:165

*GP, general practitioner; ED, emergency department.

**Figure:**
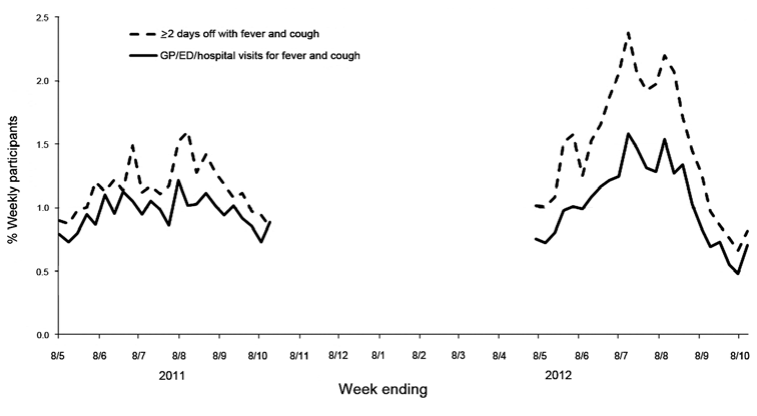
Index of severity: percentage of participants with fever and cough plus >2 days off work and participants seeking medical advice from general practitioner (GP), emergency department (ED), or hospital inpatient admission (denominator is number of weekly participants), Australia, May 2011–October 2012.

## Conclusions

The addition of questions on health-seeking behavior and laboratory testing for influenza in the Flutracking surveillance system enabled rapid construction of a surveillance pyramid during 2011 and 2012 with progressive data available for each stratum of the pyramid on a weekly basis. Such analyses generally require integration of data from multiple and disparate surveillance systems.

Every Flutracking participant who reported laboratory-confirmed influenza represented 96 to 595 cases of cough and fever in the larger cohort. Although only a proportion of cough and fever cases would be true influenza, the proportion of true cases can be estimated (*9*).

The increased index of severity of illness among Flutracking participants in 2012 compared to 2011 is contemporaneous with a change in the circulating influenza strains from the predominant influenza A(H1N1)pdm09 strain to a subtype H3N2 influenza strain and the increased severity of illness reported by national and regional surveillance systems (*3*). 

Although the Flutracking surveillance system relies on self-reports, its capacity to construct a surveillance pyramid from community ILI through to confirmed influenza and various strata of surveillance in near real-time is a unique attribute. Constructing such pyramids will facilitate the estimation of community level attack rates and severity of influenza, changes in health-seeking behavior, and influenza testing during seasonal and pandemic influenza periods.
